# Incident Atrial Fibrillation and In-Hospital Mortality in SARS-CoV-2 Patients

**DOI:** 10.3390/biomedicines10081940

**Published:** 2022-08-10

**Authors:** Alessandro Maloberti, Cristina Giannattasio, Paola Rebora, Giuseppe Occhino, Nicola Ughi, Marco Biolcati, Elena Gualini, Jacopo Giulio Rizzi, Michela Algeri, Valentina Giani, Claudio Rossetti, Oscar Massimiliano Epis, Giulio Molon, Anna Beltrame, Paolo Bonfanti, Maria Grazia Valsecchi, Simonetta Genovesi

**Affiliations:** 1 Cardiology 4, “A. De Gasperis” Cardio Center, ASST GOM Niguarda Ca’ Granda, 20162 Milan, Italy; 2 School of Medicine and Surgery, University of Milano-Bicocca, 20126 Milan, Italy; 3 Bicocca Bioinformatics, Biostatistics and Bioimaging Centre—B4, School of Medicine and Surgery, University of Milano-Bicocca, 20126 Milan, Italy; 4 Rheumatology, Multispecialist Medical Department, ASST GOM Niguarda Ca’ Granda, 20126 Milan, Italy; 5 Nuclear Medicine, ASST GOM Niguarda Ca’ Granda, 20126 Milan, Italy; 6 Cardiology Department, IRCCS Sacro Cuore Don Calabria Hospital, 37024 Negrar di Valpolicella, Italy; 7 Department of Infectious-Tropical Diseases and Microbiology, IRCCS Sacro Cuore Don Calabria Hospital, 37024 Negrar di Valpolicella, Italy; 8 Infectious Disease Department, San Gerardo Hospital, 20900 Monza, Italy; 9 Cardiology Unit, Istituto Auxologico Italiano, IRCCS, 20133 Milan, Italy

**Keywords:** incident atrial fibrillation, SARS-CoV-2, in-hospital mortality, intensive care unit

## Abstract

(1) Background: Among the different cardiovascular (CV) manifestations of the coronavirus disease 2019 (COVID-19), arrhythmia and atrial fibrillation (AF) in particular have recently received special attention. The aims of our study were to estimate the incidence of AF in patients hospitalized for COVID-19, and to evaluate its role as a possible predictor of in-hospital all-cause mortality. (2) Methods: We enrolled 3435 people with SARS-CoV2 infection admitted to three hospitals in Northern Italy from February 2020 to May 2021. We collected data on their clinical history, laboratory tests, pharmacological treatment and intensive care unit (ICU) admission. Incident AF and all-cause in-hospital mortality were considered as outcomes. (3) Results: 145 (4.2%) patients developed AF during hospitalization, with a median time since admission of 3 days (I-III quartile: 0, 12). Patients with incident AF were admitted more frequently to the ICU (39.3 vs. 12.4%, *p* < 0.001), and more frequently died (37.2 vs. 16.9%, *p* < 0.001). In the Cox regression model, the significant determinants of incident AF were age (HR: 1.041; 95% CI: 1.022, 1.060 per year), a history of AF (HR: 2.720; 95% CI: 1.508, 4.907), lymphocyte count (HR: 0.584; 95% CI: 0.384, 0.888 per 10^3^/µL), estimated glomerular filtration rate (eGFR, HR: 0.988; 95% CI: 0.980, 0.996 per mL/min) and ICU admission (HR: 5.311; 95% CI: 3.397, 8.302). Incident AF was a predictor of all-cause mortality (HR: 1.405; 95% CI: 1.027, 1.992) along with age (HR: 1.057; 95% CI: 1.047, 1.067), male gender (HR: 1.315; 95% CI: 1.064; 1.626), dementia (HR: 1.373; 95% CI: 1.045, 1.803), lower platelet (HR: 0.997; 95% CI: 0.996, 0.998 per 10^3^/µL) and lymphocyte counts (HR: 0.843; 95% CI: 0.725, 0.982 per 10^3^/µL), C-Reactive protein values (HR: 1.004; 95% CI: 1.003, 1.005 per mg/L), eGFR (HR: 0.990; 95% CI: 0.986, 0.994 per mL/min), and ICU admission (HR: 1.759; 95% CI: 1.292, 2.395). (4) Conclusions: Incident AF is a common complication in COVID-19 patients during hospitalization, and its occurrence strongly predicts in-hospital mortality.

## 1. Introduction

Coronavirus disease 2019 (COVID-19) primarily results in a respiratory disease (Severe Acute Respiratory Distress Syndrome—ARDS); through endothelial damage [1], it may also result in multi-organ involvement, including the cardiovascular (CV) system [2]. Indeed, patients with CV risk factors (diabetes mellitus, arterial hypertension, obesity) and those with established CV disease represent a vulnerable population in which COVID-19 results in increased morbidity and mortality [3].

Severe Acute Respiratory Syndrome Coronavirus-2 (SARS-CoV-2) is able to directly lead to CV complications such as acute coronary syndrome, pulmonary embolism and myocarditis. Recently, among CV complications related to COVID-19, particular attention has been paid to arrhythmia [2], and this risk is still being evaluated [4,5]. Sinus tachycardia is frequently seen in patients with COVID-19 [6,7]; a high incidence of in-hospital atrial fibrillation (AF) has also been reported [8,9,10,11,12,13]. In a meta-analysis including 21,653 patients and 19 studies, the reported prevalence of AF was 11%, and it was even higher in patients with a severe COVID-19 infection (19 vs. 3%) [9]. In the same study, incident AF was found in 10% of COVID-19-infected subjects during hospitalization [9]. Finally, both a history of AF and incident AF were found to be associated with a higher risk of in-hospital death in these patients [8,9,10,11,12,13].

The aim of our study was to assess the incidence of AF episodes in patients hospitalized for COVID-19, and to evaluate its predictors and relationship with in-hospital all-cause mortality.

## 2. Materials and Methods

### 2.1. Study Population

This multicenter study included three hospitals in Northern Italy: San Gerardo Hospital in Monza, Niguarda Hospital in Milan and Sacro Cuore Don Calabria Hospital in Negrar di Valpolicella. All of the consecutive adult patients (≥18 years of age) diagnosed with SARS-CoV-2 infection and admitted to the three centers from February 2020 (after the first Italian autochthonous COVID case on 21 February 2020) to May 2021 were included in the study. This period was chosen in order to include the first two waves of COVID-19 infection.

Data were collected from hospital case records, and patients were followed until the first occurrence of either hospital discharge, transfer to another facility, or death.

SARS-CoV-2 infection was diagnosed if the genome was detected by reverse transcriptase-polymerase chain reaction (RT-PCR) for one or more out of three SARS-CoV-2 genes tested on at least one nasopharyngeal swab.

### 2.2. Outcomes and Covariates

Incident AF and in-hospital mortality (all-cause mortality) were the main clinical outcomes. Incident AF was defined as the first onset of AF during hospitalization in patients without a previous diagnosis of permanent AF.

The diagnosis of incident AF was made according to international guidelines as an arrhythmia without P waves with an arrhythmic R-R interval. It was diagnosed either by a 12-lead electrocardiogram after the onset of the patients’ symptoms, by the identification of a non-regular pulse at the physician’s visit, or as an episode on an electrocardiogram monitoring system lasting at least 30 s.

AF episodes were identified by reviewing the electronic medical records of the three hospitals for all of the enrolled patients, looking for the term “atrial fibrillation” or “AF” in the medical record. The patient’s medical history, pharmacological treatment, hematochemical variables at admission, and admission to the intensive care unit (ICU) during hospitalization were considered as possible predictors of incident AF and in-hospital mortality. In detail, the in-patient’s electronical medical records were used to collect data on age, gender, medical history (history of AF, heart failure, Chronic Coronary Syndrome—CCS, peripheral vascular disease, previous stroke, dementia, chronic obstructive pulmonary disease, advanced liver disease, cancer and diabetes mellitus), and admission to the ICU.

Hematochemical variables included creatinine, hemoglobin, platelet and lymphocyte counts, and C-reactive protein (CRP). The glomerular Filtration Rate was estimated (eGFR) by using the Chronic Kidney Disease Epidemiology Collaboration (CKD-EPI) equation [14]. Chronic Kidney Disease (CKD) was defined as eGFR < 60 mL/min.

Drugs for AF rhythm or rate control (beta-blockers, antiarrhythmic and digoxin), renin-angiotensin-aldosterone system (RAAS) inhibitors (angiotensin converting enzyme inhibitors and angiotensin 2 receptor blockers), anticoagulants, antiplatelets, antidiabetics and statins were recorded.

### 2.3. Compliance with Ethical Standards

The study (COMORBIDITIES) was approved by the Ethics Committee of the coordinating center (San Gerardo Hospital, Monza, Italy) and by the IRB of each center, and was registered at ClinicalTrials.gov (Identifier: NCT04670094, 15 December 2020).

When possible, informed consent was obtained from the subjects involved in the study. Otherwise, this retrospective study was conducted under authorizations guaranteed by Article 89 of the GDPR EU Regulation 2016/679, which guarantees processing for the purposes of public interest, or for the scientific, historical or statistical purposes of health data.

### 2.4. Statistical Analysis

The continuous data were described by medians and interquartile ranges (I–III quartile) and compared using the Kruskal–Wallis test; alternatively, if they were normally distributed, they were described by means and standard deviations (SD), and compared using the unpaired two-sample *t*-test. Categorical data were described by counts and percentages, and were compared using the Chi-square test. The Aalen–Johansen estimator was used to estimate the crude cumulative incidence of in-hospital mortality by AF occurrence, accounting for discharge as a competing event. Due to the time-dependence of AF occurrence, the landmark approach was used: the origin time was fixed at the median time since admission of the incident AF occurrence, and the patients who developed AF were included in the risk set at the time of AF occurrence. The patients who did not develop AF were also stratified according to their history of AF before the admission.

A cause-specific Cox proportional-hazards regression model was used for the investigation of significant determinants of incident AF, while considering death and discharge as competing events. The covariates included in the regressor were established by a backward stepwise approach, starting from the model with all comorbidities, blood chemistry tests and time-dependent admission to the ICU (before AF occurrence). Admission therapies were also added in a further model. Sex, age and history of non-permanent AF were forced in the selection, and a significance level of 0.05 was used for each selection step. This model excluded patients with permanent AF, as they were not eligible for incident AF.

A Cox proportional-hazards regression model was used for the investigation of the association between AF occurrence and in-hospital mortality. Incident AF was included as a time-dependent variable in order to account for immortal-time bias. Potential confounders were established by a backward stepwise approach, starting from the model with all comorbidities, blood chemistry tests, and ICU admission directly from the emergency department. Sex and age were forced in the selection, and a significance level of 0.05 was used for each selection step.

Hazard ratios (HRs) with 95% confidence intervals (CIs) were reported. SAS 9.4 (SAS institute, Cary, NC, USA) was used for the statistical analyses, and the first type error was set at 0.05 (two-tailed).

## 3. Results

### 3.1. Incident Atrial Fibrillation

Table 1 shows the demographic and clinical characteristics of the 3435 enrolled patients, overall and stratified according to the presence of an incident AF episode during hospitalization. The median age of the patients was 65 years (I–III quartile: 53, 77), with a male prevalence of 65%.

One hundred and forty-five patients (4.2%) developed AF during hospitalization, with a median time of 3 (I–III quartile: 0, 12) days since admission. They were older (median age 73 vs. 64 years, *p* < 0.001), had a lower eGFR (62.0 ± 25.6 vs. 74.4 ± 26.4 mL/min, *p* < 0.001), and had a higher prevalence of CKD (43.0 vs. 28.3%, *p* < 0.001) than the subjects who did not experience incident AF. Furthermore, patients with incident AF had lower platelets (199.6 ± 95.4 vs. 222.9 ± 96.3 10^3^/µL, *p* = 0.005) and lymphocyte counts (0.75 (I–III quartile: 0.55, 1.07) vs. 1.03 (0.72, 1.43) 10^3^/µL, *p* < 0.001) and higher CRP values (90.0 (I–III quartile: 51.5, 168.0) vs. 63.1 (24.5, 119.0) mg/L, *p* < 0.001).

The patients with incident AF were more frequently taking beta blockers (37.8 vs. 21.1%, *p* < 0.001), antiarrhythmics (9.0 vs. 3.0%, *p* < 0.001), RAAS inhibitors (41.2 vs. 32.5%, *p* = 0.045), antiplatelets (26.2 vs. 17.8%, *p* = 0.025) and statins (18.0 vs. 11.2%, *p* = 0.031). Among the patients who were not hospitalized in an ICU, 88 (over 2981, 3%) developed AF, compared with the 57 (over 454, 12.6%) among patients who were admitted to an ICU. Among them, 48 developed AF after or on the same day of ICU admission, and nine developed AF before ICU admission.

According to the Cox multivariable regression, the significant determinants of incident AF were age (HR: 1.041; 95% CI: 1.022, 1.060), a history of non-permanent AF (HR: 2.720; 95% CI: 1.508, 4.907), and ICU admission (HR: 5.311; 95% CI: 3.397, 8.302), while lymphocyte count (HR: 0.584; 95% CI: 0.384, 0.888) and eGFR (HR: 0.988; 95% CI: 0.980, 0.996) were inversely associated with AF occurrence (Table 2). The results were confirmed when admission therapies (beta-blockers, antiarrhythmics and anticoagulants) were added to the model (Supplementary Materials Table S1).

### 3.2. All-Cause Mortality

**Figure 1:**
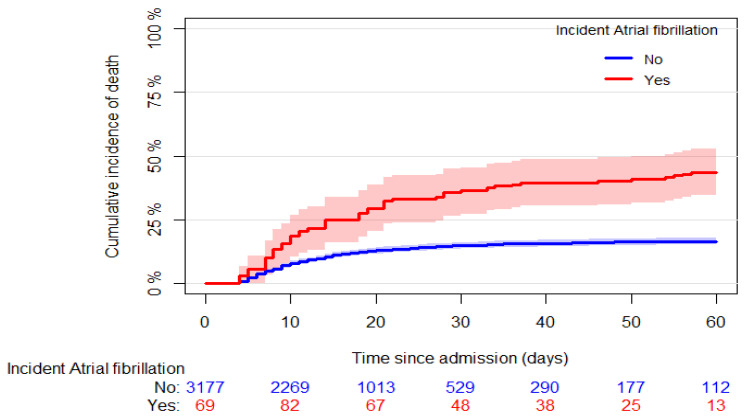
Crude cumulative incidence curves of the all-cause mortality in patients with and without incident AF. The origin was fixed at the median time of incident AF (3 days since hospital admission), and late entry was used for AF patients at the time of AF occurrence.

**Figure 2:**
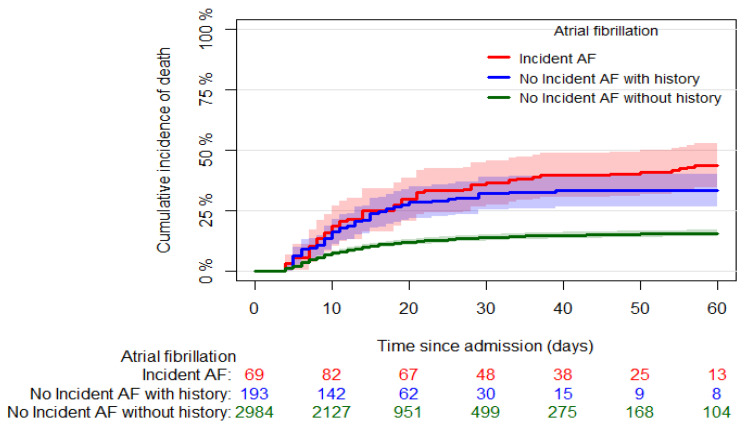
Crude cumulative incidence curves of the all-cause mortality in patients without incident AF, divided according to the presence/absence of previous AF history and patients with incident AF. The origin was fixed at the median time of incident AF (3 days since hospital admission), and late entry was used for AF patients at the time of AF occurrence.

Six hundred eleven (17.8%) in-hospital deaths were recorded; 557 out of 3290 (16.9%) were in patients without incident AF, and 54 out of 145 (37.2%) were in patients with incident AF (*p* < 0.001). Figure 1 shows the cumulative incidence of mortality in patients who developed and did not develop AF (60-day mortality: 43.6% [95% CI: 34.5, 52.7] and 16.6% [95% CI: 15.3, 17.9], respectively, *p* < 0.001). Stratifying the latter group of patients according to AF history, we found higher mortality in patients with AF history than those without (Figure 2, 60-day mortality: 33.2% [95% CI: 26.4, 40.1] and 15.5% [95% CI: 14.2, 16.9], respectively, *p* < 0.001).

According to the Cox multivariable regression, the significant determinants of all-cause mortality were age (HR: 1.057; 95% CI: 1.047, 1.067), male gender (HR: 1.315; 95% CI: 1.064; 1.626), dementia (HR: 1.373; 95% CI: 1.045, 1.803), CRP value (HR: 1.004; 95% CI: 1.003, 1.005), ICU admission directly from the emergency department (HR: 1.759; 95% CI: 1.292, 2.395), and incident AF during hospitalization (HR: 1.405; 95% CI: 1.027, 1.992). The platelet (HR: 0.997; 95% CI: 0.996, 0.998) and lymphocyte count (HR: 0.843; 95% CI: 0.725, 0.982) and eGFR value (HR: 0.990; 95% CI: 0.986, 0.994) were inversely related with mortality (Table 3). In the multivariable model, a previous history of AF was no longer significantly associated with mortality.

## 4. Discussion

The main findings of our study were (i) incident AF complicates 4.2% of the COVID-19 infection hospitalizations, and increases significantly to 12.6% when considering only patients admitted to the ICU; (ii) incident AF was independently predicted by age, previous episodes of non-permanent AF and ICU admission; and (iii) incident AF independently predicted in-hospital mortality. Interestingly, although ICU admission was a strong predictor of mortality in COVID-19 patients, incident AF maintains an independent association with the risk of death.

### 4.1. Incident AF in COVID-19 Patients

Previous studies showed that incident AF is the most relevant arrhythmia complicating hospitalization by COVID-19. In one of the largest studies on arrhythmia related to COVID-19 infection [15], AF was found to complicate 3.5% of the hospitalizations (17.7% in patients admitted to ICU). Similar data were found by other authors, who showed an incidence of between 2.5% and 7.5% [16,17]. When studies focused only on ICU inpatients, the incident of AF ranged from 6.7% to 14.9% [13,18].

In our population, beside the expected impact of age and previous AF history, the strongest predictor of incident AF was ICU admission. The association between incident AF and ICU admission is not only related to COVID-19 infection [19]. In fact, previous data showed that, in patients with ARDS from various origins (pneumonia, sepsis, aspiration and trauma), new-onset AF arose in 10% of cases [20]. In septic patients, 23% experienced incident AF, with an increase in frequency correlating with the severity of sepsis (10% in sepsis, 22% in severe sepsis, and 40% in septic shock) [21]. In addition, a high rate of incident AF (7%) was found in a large database (138,722 patients) of sepsis survivors [22]. In these studies, but also in others, new-onset AF was related to in-hospital and/or short-term mortality [20,21,22,23]. The same findings were also confirmed by meta-analysis in terms of both prevalence and a strong relationship with mortality [24,25,26,27,28].

Many factors contribute to the development of AF in critically ill patients: hypoxaemia, altered serum potassium values, the use of vasopressor/inotropes, acute kidney injury, and a systemic inflammatory response that increase levels of circulating stress hormones levels and activates the sympathetic nervous system [22]. All of these factors are also present in COVID-19 patients, particularly in the most critically ill. Serum potassium derangements are common in COVID-19 patients [29], and both the presence of CKD and incident acute kidney injury have been associated with incident AF in these individuals [30]. Furthermore, the severity of inflammation (assessed as the leukocyte count, CRP, erythrocyte sedimentation rate and procalcitonin levels) and hypoxaemia [31,32] have also been confirmed to be related to incident AF in COVID-19 patients.

Some specific features related to COVID-19 infection have also been hypothesized [33]. Angiotensin Converting Enzyme (ACE) 2 receptor is internalized after binding with SARS-CoV-2, resulting in the entry of the virus into the cell, and also resulting in a reduction of ACE2 receptors on the cell surface. This leads to a reduction in the degradation of angiotensin II to cardioprotective angiotensin 1–7 (Ang1–7), with an end effect that induces cardiac hypertrophy, vasoconstriction, tissue fibrosis and increased oxidative stress [34]. All of these are factors that may increase the susceptibility to AF. Similarly, the virus interacts with the extracellular matrix metalloproteinase inducer (a transmembrane glycoprotein), leading to the upregulation of the expression of several cytokines and promoting oxidative stress [35]. Finally, the specific role of SARS-CoV-2 in determining endothelial dysfunction has been characterized well [1].

### 4.2. Incident AF and In-Hospital Death in COVID-19 Patients

We found that incident AF was strongly associated with death. In our population, ICU admission (HR: 1.759; 95% CI: 1.292, 2.395) and incident AF (HR: 1.405; 95% CI: 1.027, 1.992) were both independent determinants of in-hospital mortality.

A trend toward increased mortality in COVID-19 patients who develop AF has been described [9]. However, only in four studies did this association persist after correction for possible confounding factors [13,32,36,37]. Peltzer et al. [32] described 9.6% (101 cases) incident AF in a population of 1053 patients with severe COVID-19 infection. They found age, male sex, previous AF, CKD and hypoxia to be determinants of incident AF (similarly to our data), along with an increased risk of death determined independently by incident AF (OR: 2.87; 95% CI: 1.33, 3.52). The multivariable model was adjusted by age, gender, race, body mass index, comorbidities (CCS, heart failure, prior stroke, history of AF, hypertension, lung disease, renal disease, cancer) and therapies (immunosuppression, RAAS blockers, nonsteroidal inflammatory, proton pump inhibitor and statins), while only hypoxia was used as a marker of disease severity.

A second study [13] was performed in a population of 9564 patients hospitalized for COVID-19 infection, 1109 (11%) of whom underwent AF. Using a propensity score matching analysis, the authors found a HR for death of 1.56 (95% CI: 1.42, 1.71) in patients with incident AF compared to patients without. In this study, CRP, D-dimer and lactate values were used as markers of disease severity.

In a small study (175 COVID-19 infected patients with only 25 new-onset AF) published recently [36], similar results were found after adjustment for respiratory failure (defined as the PaO_2_/FiO_2_ ratio) and Interleukin−6 as markers of disease severity. Interestingly, the latter marker was very high in subjects with incident AF, and was independently related to death in the multivariable analysis.

Finally, Sano et al. [37] found an association between incident AF (28 subjects over 673 total subjects, 4.1%) and mortality (OR: 4.71; 95% CI: 1.63, 13.6) in a multivariable model that included age, sex, the number of comorbidities, respiratory rate, oxygen saturation, urea and CRP.

Other studies failed to demonstrate an association between incident AF and mortality [15,17,38,39]. However, these studies were performed on small samples of patients with very few cases of incident AF, which is probably the reason for the lack of significant association.

### 4.3. Limitations and Strengths

Our study has some limitations, the main one being the absence of post-discharge follow-up, which prevents us from defining the long-term effect on mortality of incident AF. Second, although the study is multicentric, the data are from North Italy only; therefore, they may reflect regional and local health policies, and might not be generalizable to all hospitals or all countries. In addition, only admitted patients were analyzed. It is likely that the incidence of AF in COVID-19 outpatients is lower than that in inpatients, who are surely more severely affected by the disease. However, the evaluation of incident AF in the outpatient setting is very difficult; in fact, to the best of our knowledge, there is no study on COVID-19 patient populations performed in this setting.

Third, not all of our hospitalized patients with COVID-19 were undergoing continuous electrocardiographic monitoring (especially those not admitted to the ICU); therefore, it is possible that asymptomatic incident AF episodes could not be detected, resulting in an underestimation of the phenomenon.

Fourth, the enrollment period of the study mainly involves patients infected by the native and alpha variants. After that period, other variants with a more infectious but less severe disease became dominant. Because, according our hypothesis, incident AF is mainly related to disease severity, the inclusion of a less severe form could lead to a lower frequency of incident AF. This is also possible for future variants, for which the risk of incident AF, and its prognostic role, should be reevaluated.

Finally, some important covariates were not systematically collected and, thus, cannot be used for the present analysis. Among them, some echocardiographic (left ventricular ejection fraction, left atrial dimension, pericardial effusion) and biochemical (particularly natriuretic peptides and troponin) variables have been identified as important prognostic markers in SARS-CoV-2-infected patients [40,41,42] but, unfortunately, were not systematically available in our dataset. Due to the fact that these evaluations were performed only on patients with severe infection (by clinical practice), the inclusions of these variables in the models would lead a selection bias.

In addition, other factors, such as the treatment the patients underwent during hospitalization and the patients’ socioeconomic status were not considered in the analysis. Details on how AF incident episodes were managed are also not available (pharmacological or electrical cardioversion vs. the spontaneous restoration of rhythm or the management of rhythm control); however, this does not impact our conclusions on the incidence of AF, although it might influence mortality. Despite these limitations, our study presents some strengths compared with previously published studies. First, although COVID-19 disease severity is not universally defined (and in fact several variables have been used in the multivariable model to correct for sepsis severity), we included ICU admission as an unequivocal marker of disease severity. In studies prior to the COVID-19 pandemic, ICU admission was found to be a significant determinant of the onset of AF in septic patients [20], and it was associated with in-hospital and short-term mortality [21,22,23,24]. Furthermore, it has been found to be a determinant of AF [8] and death in patients infected with SARS-CoV-2 [43,44]. Therefore, our results confirm the important role of incident AF in the identification of patients with a bad in-hospital outcome, even independent of this very important prognostic factor.

The second strength of our study is the sample size in terms of both the total number of subjects (3435) and of incident AF cases (145). Finally, for the first time, we appropriately modeled the time-dependence of AF onset and ICU admission in this setting. In previous studies [13,32,36,37], all of the events occurring during hospitalization were considered simultaneous, losing the valuable information from the temporal sequence of events. Specifically, in the modeling of predictors of incident AF, we included only ICU admissions that preceded the onset of AF. Moreover, we accounted for the possible immortal-time bias on the mortality model by including AF occurrence as a time-dependent variable.

## 5. Conclusions

In conclusion, incident AF frequently affects patients with COVID-19 during hospitalization. Whether SARS-CoV-2 has a specific role in causing incident AF or it is mainly related to ARDS and severe sepsis need to be better elucidated in future studies. However, incident AF is strongly related to in-hospital mortality, even adjusting for ICU admission; therefore, incident AF should be taken into account when considering the mortality risk of patients hospitalized for COVID-19 infection.

## Figures and Tables

**Table 1:** Clinical characteristics of the included patients, overall and divided according to in-hospital incident AF occurrence.

		Overall	Incident AF = No	Incident AF = Yes	*p*	Missing (%)
n		3435	3290	145		
Demographic variables						
Male, n (%)		2233 (65.0)	2129 (64.7)	104 (71.7)	0.1	0
Age (years) (median (I–III quartiles))		65 (53, 77)	64 (53, 76)	73 (64, 81)	<0.001	0
Anamnestic variables						
Time to incident AF (days) (median (I–III quartiles))		3 (0, 12)	-	30 (0, 12)	-	0
History of AF, n (%)		218 (6.3)	203 (6.2)	15 (10.3)	0.065	0
Non-permanent AF, n (%)		101 (46.3)	86 (42.3)	15 (100.0)	<0.001	0
Permanent AF, n (%)		117 (53.7)	117 (57.6)	0 (0.0)	-	0
Chronic Coronary Syndrome, n (%)		314 (9.2)	299 (9.1)	15 (10.3)	0.733	0.6
Heart failure, n (%)		160 (4.7)	148 (4.5)	12 (8.3)	0.059	0.6
Peripheral vascular disease, (%)		226 (6.6)	215 (6.6)	11 (7.6)	0.759	0.6
Previous stroke, n (%)		287 (8.4)	270 (8.3)	17 (11.7)	0.188	0.6
Dementia, n (%)		235 (6.9)	224 (6.9)	11 (7.6)	0.862	0.6
COPD, n (%)		298 (8.7)	283 (8.7)	15 (10.3)	0.579	0.6
Advanced liver disease, n (%)		133 (3.9)	131 (4.0)	2 (1.4)	0.17	0.8
Cancer (%)		341 (10.1)	326 (10.0)	15 (10.4)	0.998	1.3
Diabetes mellitus, n (%)		584 (17.1)	554 (17.0)	30 (20.7)	0.291	0.6
Biochemical variables						
Creatinine (mg/dL) (mean (SD))		1.17 (1.03)	1.16 (1.02)	1.42 (1.19)	0.005	6.6
eGFR (mL/min) (mean (SD))		73.9 (26.4)	74.4 (26.4)	62.0 (25.6)	<0.001	6.6
CKD (eGFR < 60 mL/min), n (%)		929 (28.9)	871 (28.3)	58 (43.0)	<0.001	6.6
Hemoglobin (g/dL) (mean (SD))		12.7 (1.9)	12.7 (1.9)	12.4 (2.0)	0.08	4.5
Platelet (10^3^/µL) (mean (SD))		221.9 (96.4)	222.9 (96.3)	199.6 (95.4)	0.005	4.5
Lymphocytes (10^3^/µL) (median (I–III quartiles))		1.01 (0.70, 1.42)	1.03 (0.72, 1.43)	0.75 (0.55, 1.07)	<0.001	7
C Reactive Protein (mg/L) (median (I–III quartiles))		64.2 (25.0, 121.6)	63.1 (24.5, 119.0)	90.0 (51.5, 168.0)	<0.001	11.2
Therapies						
RAAS inhibitors, n (%)		1050 (32.9)	994 (32.5)	56 (41.2)	0.045	7.1
Beta blockers, n (%)		731 (21.8)	677 (21.1)	54 (37.8)	<0.001	2.6
Antiarrhythmic, n (%)		108 (3.2)	95 (3.0)	13 (9.0)	<0.001	2.5
Digoxin, n (%)		15 (0.5)	15 (0.5)	0 (0.0)	0.907	9.4
Anticoagulant, n (%)		360 (10.7)	351 (10.9)	9 (6.3)	0.111	1.8
Antiplatelet, n (%)		567 (18.2)	535 (17.8)	32 (26.2)	0.025	9.2
Antidiabetic, n (%)		422 (13.4)	404 (13.4)	18 (14.8)	0.765	8.6
Statins, n (%)		361 (11.5)	339 (11.2)	22 (18.0)	0.031	8.6
Hospitalization variables						
Hospital length (days) (median (I–III quartiles))		13 (8, 22)	12 (7, 21)	22 (13, 41)	<0.001	0
ICU admission, n (%)		454 (13.6)	397 (12.4)	57 * (39.3)	<0.001	2.7
Time to ICU admission (days) (median (I–III quartiles))		1 (0, 4)	1 (0, 4)	4 (0, 7)	0.003	0.9
Outcome						
All-cause death, n (%)		611 (17.8)	557 (16.9)	54 (37.2)	<0.001	0

Abbreviation: AF = Atrial Fibrillation; COPD = Chronic Obstructive Pulmonary Disease; eGFR = estimated Glomerular Filtration Rate; CKD = Chronic Kidney Disease; RAAS = Renin Angiotensin Aldosterone System; ICU = Intensive Care Unit; ED = Emergency Department. * Of which 48 were admitted to the ICU before or at AF occurrence (21 had ICU admission directly from ED), and 9 were admitted to the ICU after AF occurrence.

**Table 2:** Cox multivariable regression model on incident AF with stepwise backward variable selection (variables with *p* < 0.05 were retained; age, sex and history of AF were forced in the model). Patients with permanent AFs were excluded.

Number = 2725; Incident AF = 121				
Parameter	HR	95% Confidence Interval		*p*-Value
Male (yes vs. no)	1.217	0.806	1.838	0.3497
Age (years)	1.041	1.022	1.060	<0.0001
History of non-permanent AF (yes vs. no)	2.720	1.508	4.907	0.0009
Lymphocytes (10^3^/µL)	0.584	0.384	0.888	0.0119
eGFR (mL/min)	0.988	0.980	0.996	0.0019
ICU admission (yes vs. no-time dependent)	5.311	3.397	8.302	<0.0001

Abbreviation: AF = Atrial Fibrillation; eGFR = estimated Glomerular Filtration Rate; ICU = Intensive Care Unit.

**Table 3:** Cox multivariable regression model on death with stepwise backward variable selection (variables with *p* < 0.05 were retained; age and sex were forced in the model).

Number = 2755; Death = 472.				
Parameter	HR	95% Confidence Interval		*p*-Value
Male (yes vs. no)	1.315	1.064	1.626	0.0114
Age (years)	1.057	1.047	1.067	<0.0001
Dementia (yes vs. no)	1.373	1.045	1.803	0.0228
Platelet (10^3^/µL)	0.997	0.996	0.998	<0.00001
Lymphocytes (10^3^/µL)	0.843	0.725	0.982	0.0279
CRP (mg/L)	1.004	1.003	1.005	<0.0001
eGFR (mL/min)	0.990	0.986	0.994	<0.0001
Incident AF (yes vs. no-time dependent)	1.405	1.027	1.922	0.0333
ICU admission directly from ED (yes vs. no)	1.759	1.292	2.395	0.0003

Abbreviation: CRP = C-Reactive Protein; eGFR = estimated Glomerlar Filtration Rate; AF = Atrial Fibrillation; ICU = Intensive Care Unit; ED = Emergency Department.

## Data Availability

Available on request.

## Supplementary Materials

The following supporting information can be downloaded at https://www.mdpi.com/article/10.3390/biomedicines10081940/s1. Table S1 caption: Cox regression model for incident AF with stepwise backward variable selection, including admission therapies (variables with *p* < 0.05 + age, sex, history of AF and antiarrhythmics. Permanent AFs were excluded).
