# Supplementary material for: Incident Atrial Fibrillation and In-Hospital Mortality in SARS-CoV-2 Patients
Source: Biomedicines. 2022 Aug 10;10(8):1940. doi: 10.3390/biomedicines10081940 (PMC9406191; doi:10.3390/biomedicines10081940)
Supplement: Supplementary file 1 [file biomedicines-10-01940-s001.zip › biomedicines-1772817-supplementary.pdf]

Table S1: Cox regression model for incident AF with stepwise backward variable selection including admission therapies (variables with p<0.05 + age, sex, history of AF and antiarrhythmic. Permanent AFs were excluded).

**Number = 2326; incident AF = 91**				
**Parameter**	**HR**	**95% confidence interval**		**P-value**
Male (yes vs no)	1.205	0.752	1.933	0.4384
Age (years)	1.036	1.015	1.058	0.0007
History of AF (yes vs no)	2.450	1.143	5.248	0.0212
Antiarrhythmic (yes vs no)	1.786	0.807	3.952	0.1525
Lymphocytes (10^3/µL)	0.472	0.282	0.790	0.0043
eGFR (mL/min)	0.988	0.980	0.997	0.0099
ICU admission during hospitalization (yes vs no - time dependent)	4.627	2.730	7.843	<0.0001
Beta blockers (yes vs no)	1.615	1.036	2.518	0.0342
Anticoagulant (yes vs no)	0.350	0.146	0.841	0.0189

Abbreviation: AF = Atrial Fibrillation; eGFR = estimated Glomerual Filtration Rate; ICU = Intensive Care Unit.
